# Maternal Mercury Exposure and Hypertensive Disorders of Pregnancy: A Systematic Review

**DOI:** 10.1055/s-0042-1760215

**Published:** 2022-12-29

**Authors:** Aline de Oliveira Dantas, Thiania dos Santos da Silva de Castro, Volney de Magalhães Câmara, Aline de Souza Espindola Santos, Carmen Ildes Rodrigues Froes Asmus, Angelica dos Santos Vianna

**Affiliations:** 1 Faculdade de Medicina, Universidade Federal do Rio de Janeiro, Rio de Janeiro, RJ, Brazil

**Keywords:** mercury, pregnancy-induced hypertension, preeclampsia, eclampsia and gestational hypertension, mercúrio, hipertensão induzida pela gestação, pré-eclâmpsia, eclâmpsia e hipertensão gestacional

## Abstract

**Objective** The present review aimed to synthesize the evidence regarding mercury (Hg) exposure and hypertensive disorders of pregnancy (HDP).

**Data Sources** The PubMed, BVS/LILACS, SciELO and UFRJ's Pantheon Digital Library databases were systematically searched through June 2021.

**Study Selection** Observational analytical articles, written in English, Spanish, or Portuguese, without time restriction.

**Data Collection** We followed the PICOS strategy, and the methodological quality was assessed using the Downs and Black checklist.

**Data Synthesis** We retrieved 77 articles, of which 6 met the review criteria. They comprised 4,848 participants, of which 809 (16.7%) had HDP and 4,724 (97.4%) were environmentally exposed to Hg (fish consumption and dental amalgam). Mercury biomarkers evaluated were blood (four studies) and urine (two studies). Two studies found a positive association between Hg and HDP in the group with more exposure, and the other four did not present it. The quality assessment revealed three satisfactory and three good-rated studies (mean: 19.3 ± 1.6 out 28 points). The absence or no proper adjustment for negative confounding factor, such as fish consumption, was observed in five studies.

**Conclusion** We retrieved only six studies, although Hg is a widespread toxic metal and pregnancy is a period of heightened susceptibility to environmental threats and cardiovascular risk. Overall, our review showed mixed results, with two studies reporting a positive association in the group with more exposure. However, due to the importance of the subject, additional studies are needed to elucidate the effects of Hg on HDP, with particular attention to adjusting negative confounding.

## Resumo

**Objetivo** A presente revisão busca sintetizar as evidências em relação à exposição ao mercúrio (Hg) e os distúrbios hipertensivos da gestação (DHG).

**Fontes Dos Dados** Os bancos de dados PubMed, BVS/LILACS, SciELO e a Biblioteca Digital da UFRJ Pantheon foram sistematicamente pesquisadas durante junho de 2021.

**Seleção de estudos** Artigos observacionais analíticos, escritos em inglês, espanhol ou português, sem restrição temporal.

**Coleta de Dados** A estratégia PICOS foi seguida e a qualidade metodológica foi avaliada usando o checklist Downs and Black.

**Síntese de dados** Foram encontrados 77 artigos, dos quais 6 atenderam aos critérios da revisão. Foram 4.848 participantes, dos quais 80 (16,7%) tinham DHG e 4.724 (97,4%) estavam expostos ambientalmente ao Hg (consumo de peixe e amálgama dental). Os biomarcadores de mercúrio avaliados foram sangue (quatro estudos) e urina (dois estudos). Dois estudos encontraram associação positiva entre Hg e DHG no grupo com maior exposição e os outros quatro não a apresentaram. A avaliação de qualidade metodológica revelou 3 estudos satisfatórios e 3 bons (média: 19,3 ± 1,6 em 28 pontos). A ausência ou não de ajuste adequado para fator de confusão negativo, como consumo de pescado, foi observada em cinco estudos.

**Conclusão** Recuperamos apenas seis estudos, embora o Hg seja um metal tóxico generalizado e a gravidez seja um período de maior suscetibilidade a ameaças ambientais e risco cardiovascular. No geral, nossa revisão mostrou resultados mistos, com dois estudos relatando associação positiva no grupo com maior exposição. No entanto, devido à importância do assunto, estudos adicionais são necessários para elucidar os efeitos do Hg sobre DHG, com atenção especial ao ajuste de confundimento negativo.

## Introduction

Systemic arterial hypertension (SAH) is a highly prevalent health issue worldwide, leading to significant morbidity and costs for health systems. 1 It is equally an important public health issue during pregnancy and deserves special attention since it is one of the leading causes of maternal and perinatal mortality worldwide. 2 Besides, the traditional risk factors for SAH, including overweight/obesity, age > 60 years old, daily ingestion of sodium > 2 g, and sedentarism, multifetal pregnancy, primigravid women, and multiparas > 35 years old are additional factors for hypertensive disorders of pregnancy (HDP). 1 3 4

Environmental exposure to heavy metals, such as mercury (Hg), have been associated with adverse cardiovascular effects, including changes in blood pressure levels. 5 6 7 8 9 10 Although the mechanisms by which Hg may induce hypertension are not yet fully elucidated, some evidence points to an increase in angiotensin-converting enzyme activity, stimulation of the proliferation of vascular smooth muscle cells, induction of renal dysfunction, and an imbalance of the redox system, with an increase in oxidative stress and consequent reduction in nitric oxide bioavailability, endothelial dysfunction, and decreased smooth muscle relaxation. 6 8 11 Also, Hg can accumulate in the placenta tissue and leads to its dysfunction. 9

Mercury is a ubiquitous environmental toxic substance with adverse results for health. 10 There are three distinct forms of Hg: elemental mercury (Hg ^0^ ), inorganic mercury (IHg), and organic mercury (ethylmercury [ethylHg], methylmercury [MeHg]). Its main sources of exposure include gold mining, Chlor-alkali industry, biomass burning, and deforestation, dentist activities (Hg ^0^ ), presence of dental amalgams, skin cosmetics use (IHg), vaccines conservative (ethylHg), and fish and shellfish intake (MeHg). 12 13 14 15

The association between Hg exposure and hypertension has produced inconsistent findings. 16 Differences in study populations, and exposure levels, different Hg species, Hg biomarkers used to assess the exposure and absence of proper adjustment for confounding factors may contribute to the discrepancies observed in studies. 8

Considering the widespread distribution of Hg, the great impact of HDP on public health, and the controversial evidence about their association, the present systematic review aimed to address this topic.

## Methods

We followed the guidelines of the Preferred Reporting Items for Systematic Reviews and Meta-Analyses (PRISMA) to conduct and report the present review. 17 In addition, the study protocol was submitted to the International Prospective Register of Systematic Reviews (PROSPERO), approved under number CRD42022297367.

A search strategy was developed in three electronic databases (BVS/LILACS, PubMed/Medline, and SciELO) and one Digital Library of Theses and Dissertations (Pantheon – Universidade Federal do Rio de Janeiro) in June 2021. We used various combinations of MeSH descriptors associated with the text words: *mercury* AND *hypertension, pregnancy-induced* *hypertensive disorders of pregnancy* OR *preeclampsia* OR *eclampsia* OR *gestational hypertension* .

Articles were considered for inclusion based on the PICOS strategy, as follows: *P* articipants comprised pregnant or puerperal women; *I* ntervention included assessment of Hg exposure through its measurement in any biological matrix; *C* omparison with normotensive pregnant or puerperal women and documented Hg measurement in any biological matrix; *O* utcome comprised gestational hypertension syndromes with their criteria reported by the authors. *S* tudy: original observational analytical article, written in Spanish, English, or Portuguese, without time restriction. We excluded any article without Hg exposure assessment in a biological matrix, without the criteria used to classify HDP, editorial articles, author's opinions, books, case reports, experimental studies (animal and in vitro); and reviews. The PubMed database was the reference database for cases of duplicate articles.

Two reviewers (Dantas A. O. and Castro T. S. S.) independently assessed the entire study selection process. Any disagreements about study selection were resolved by discussion and, if necessary, a third reviewer (Vianna A. S.) was consulted. The flowchart started by analyzing the titles, followed by the abstract, and later by the full text. Finally, we checked the reference lists of eligible papers to identify additional relevant studies.

One reviewer (Dantas A. O.) extracted the data from the eligible studies using a form that included: 1. Study characteristics: name of the first author, year of publication, country of study; 2. Methods: design, sample size, and exposure site; 3. gestational hypertension (GH) cases: number of cases, age, ethnicity; 4. Hg exposure: source, biological matrix, laboratory technique; 5. Statistical analysis including parametric (Student *t* -test and analysis of variance [ANOVA]) and nonparametric tests (Mann-Whitney and Kruskal-Wallis) for comparison (mean difference), regression tests for measure of association (risk ratio, odds ratio [OR] and/or hazard ratio), and prevalence ratio; 6. Methodological quality score. Another reviewer (Vianna A. S.) checked this step.

Two reviewers (Dantas A. O. and Castro T. S. S.) independently assessed the quality of each eligible study according to the Downs and Black (DB) checklist. It contains 27 items, subdivided into 5 sub-scales, which assess reporting (9 items), external validity (3 items), internal validity (bias and confounding – 13 items), and power (1 item). The 25-item score is: yes = 1, no = 0 and unable to determine = 0. Item 5 (distribution of main confounding variables) presents the score: yes = 2; partially = 1 and not = 2. 18 Item 27 (power) was modified, scoring yes or no for the power calculation. We adopted the categorization of quality proposed by Hooper et al.: excellent (26–28), good (20–25), satisfactory (15–19), or poor (≤14). 19

Although we had originally planned to perform a quantitative meta-analysis, we considered it inappropriate due to methodological limitations of the selected articles and to the high heterogeneity in exposure assessment with different cutoffs. Therefore, we reported the findings as a systematic qualitative review.

## Results

**Fig. 1:**
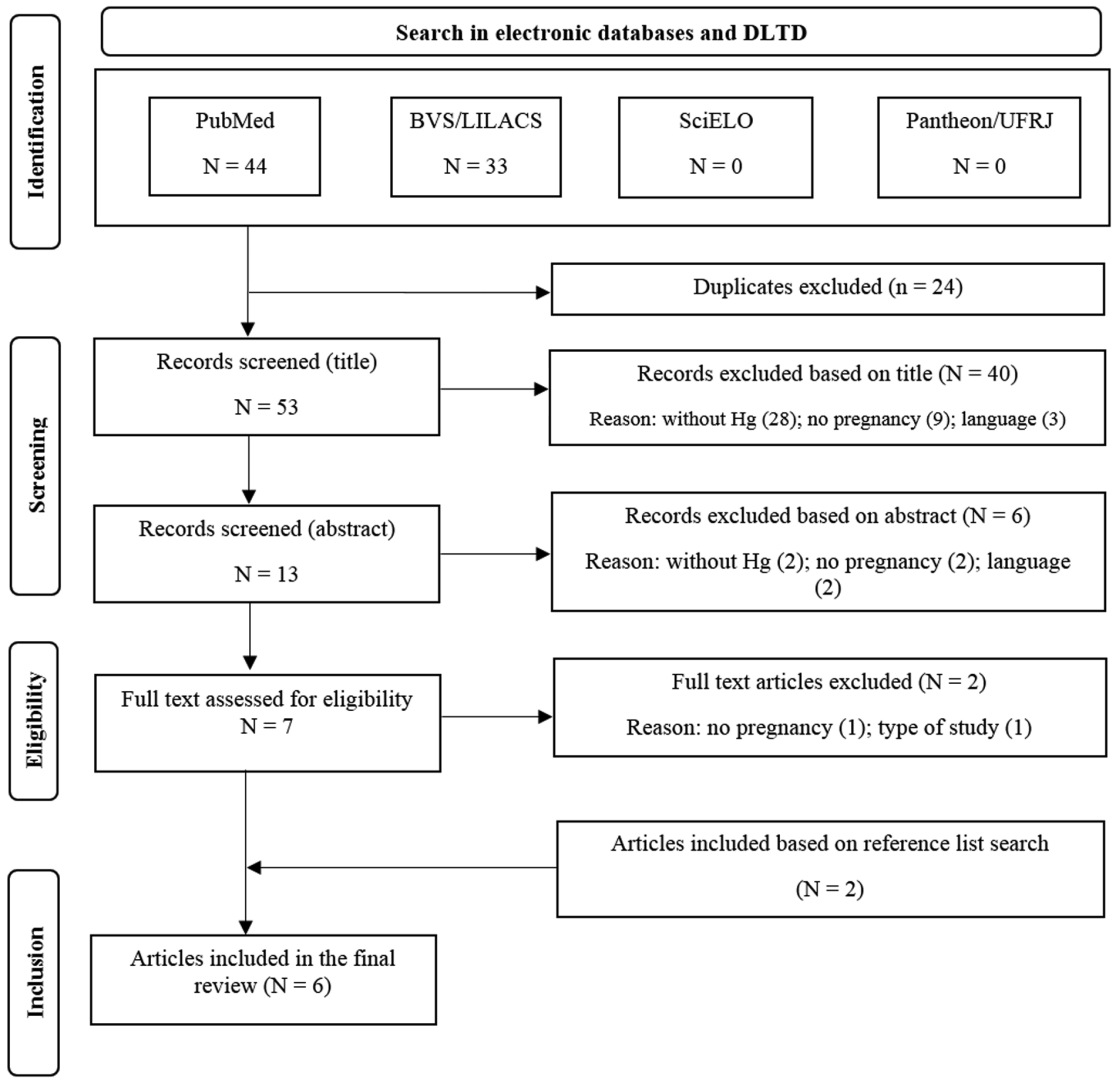
PRISMA flowchart of the study selection process. Abbreviations: DLTD, Digital Library of Theses and Dissertations; BVS/LILACS, Biblioteca Virtual em Saúde/Literatura Latino-Americana e do Caribe em Ciências da Saúde; SciELO, Scientific Library Online; UFRJ, Universidade Federal do Rio de Janeiro

The present systematic review retrieved 77 potentially eligible studies. Of these, 6 met our inclusion criteria, 4 from the electronic databases and 2 from the manual reference consultation, published between 2006 and 2020. The main reason for exclusion was out of scope, comprising 42 articles (30 without Hg and 12 without pregnant women). A flowchart of the search and screening process is displayed in Fig. 1 .

The six studies had the following design: three were cohorts and three were case control. They covered 4,848 participants from 5 countries, 3 conducted in North America (2 in the USA and 1 in Canada), 2 in Asia, and 1 in North Africa. Four studies comprised 4,724 participants (97.4%) involved primarily in environmental exposure to Hg, and 1 study with 124 participants (2.6%) had both environmental and occupational exposure. 20 21 22 23 24 25

Out of 4,848 participants, 4,039 were controls (2,514 pregnant women and 1,525 postpartum), and 809 (16.7%) had a HDP diagnosis, comprising 187 (23.1%) GH, and 622 (76.9%) preeclampsia (PE).

The participants had the following characteristics: age ranging between 15 and 49 years old, 406 (8.4%) were smokers, and 2.685 (55.4%) reported their ethnicity, with 1,794 (66.8%) white individuals.

Regarding the source of exposure, half of the studies reported it as follows: amalgam use (64 dentists), presence of dental amalgam (905 participants) and fish intake (1,817 individuals). 23 25 Concerning the latter, one study (1,817 participants) reported the frequency, but not the type of fish consumed. 23 Four studies measured Hg concentrations in blood (whole maternal blood [three], umbilical cord blood [one], and/or red blood cell [one]), and two in urine. The laboratory method more frequently used was inductively coupled plasma mass spectrometry (ICP-MS). 20 21 22 23 24 The detection limit was described in three studies, ranging from 0.12 to 0.33 μg/l (total Hg). No study investigated the association with hypertension according to the type of Hg. In addition, four studies measured other toxicants (metals) during the research. 20 21 22 24 Two studies investigated the association between the metal mixture and HDP (PE). 21 24

Statistical analysis of studies included mean difference (two studies no and the other two yes), and measures of association. 20 22 23 25 Concerning the latter, two studies reported positive association (more exposed group [Hg _urine_ = 41.8μg/g]: RR = 3.67; 95%CI = 1.25–10.76 and more exposed group [Hg _blood_ ≥1.89μg/L]: aOR multi-metal = 1.60; 95%CI = 1.08–2.38; *p* = 0.039), and the other four studies found no association (One unit increase p > 0.05; HR single model = 0.90; 95%CI = 0.63–1.28; HR (As, Hg and Sn) = 0.75; 95%CI = 0.39–1.46; Prevalence ratio = 1.03; 95%CI = 0.88–1.20; *p* = 0.71; 1 to 4 dental amalgams group: aOR = 1.31; 95%CI = 0.92–1.85 or ≥ 5 dental amalgams group - aOR = 1.32; 95%CI = 0.86, 2.04). 20 21 22 23 24 25 Regarding the latter, although the authors did not observe any association with HDP, they reported an inverse association with systolic blood pressure (dental amalgam replacement group: β = - 1.58; 95%CI = - 2.95–- 0.02; *p* = 0.02). 23 The characteristics of all six studies are summarized in Chart 1 .

**Chart 1:** Characteristics of the selected studies

Author year country	Study characteristics design; number	Characteristics of the participants		Hg assessment			Outcome	Mean difference/ measure of association
		Age (Mean: years old)	Ethnicity	Site of exposure (source)	Biological matrix (laboratory technique)	Mercury level		
Vigeh et al. (2006), 20 Iran	Case-control; *n* = 396	27	396 NI	Environ	Maternal blood and UBC (ICP-MS)	Maternal blood: PE: 1.35 (0.74); Control: 1.34 (1.19) UBC: PE: 1.69 (1.19) Control: 1.70 (1.33)	PE	Mean difference: no
El-Badry et al. (2018), 25 Egypt	Cohort; *n* = 124	Ex:25.6 NEx: 25.9	124 NI	Environ (fish) Occupat (dentist)	Urine (CVAAS)	Urine 3rd quarter: Ex: 42.8 (13.7); NEx: 7.1 (3.9)	PE	Mean difference: yes; RR _Ex_ = 3.67 (1.25–10.76)
Bommarito et al. (2019), 21 USA	Case-control; *n* = 383	32.7	231 white 57 black 95 NI	Environ	Urine (ICP-MS)	Urine 3rd quarter: PE: 0.50 (0.24, 0.76); Control: 0.51 (0.27, 0.97)	PE	HR unimetal = 0.90 (0.63 - 1.28; *p* = 0.55); HR multimetal As, Hg, Sn = 0.75 (0.39–1.46; *p* = 0.40)
Liu et al. (2019), 22 USA	Cohort; *n* = 1.274	27.99	739 black 535 NI	Environ	Maternal red blood cells (ICP-MS)	Maternal blood: PE: 2.1 (1.0–4.7); Control: 2.0 (1.0–3.6)	PE	Mean difference: no; Prevalence ratio = 1,03 (0.88–1.20; *p* = 0.71)
Louopou et al. (2020), 23 Canada	Cohort; *n* = 1.817	31.86	1.563 white 254 NI	Environ (fish, amalgam)	Maternal blood (ICP-MS)	Maternal blood 1st trimester: zero amalgams: 0.58 1 to 4 amalgams: 0.74 ≥ 5 amalgams: 0.90	GH	Mean difference: yes; aOR = 1.31 (0.92, 1.85) < 5 dental amalgams group; aOR = 1.32 (0.86, 2.04) ≥ 5 dental amalgams group
Wang et al. (2020), 24 China	Case-control; *n* = 854	20–30	854 NI	Environ	Maternal blood (ICP-MS)	Maternal blood: PE: 1.52 (0.97–2.36); Control: 1.49 (0.96–2.08)	PE	aOR = 1,60 (1,08–2,38; *p* = 0,039) in high Hg ≥ 1,89

Abbreviations: aOR, adjusted odds ratio; As, arsenic; CVAAS, cold vapor atomic absorption spectroscopy; Environ, environmental; Ex, exposed; GH, gestational hypertension; Hg, mercury; ICP-MS, inductively coupled plasma mass spectrometry; NEx, not exposed; NI, not informed; Occupat, occupational; PE, preeclampsia; Sn, tin; UBC, umbilical cord blood.

The assessment of the methodological quality of the articles by the Downs and Black checklist showed that 3 were considered satisfactory and three were rated as good (mean = 19.3 ± 1.6 out of 28 points). The representativeness of the samples and the adjustment for confounding factors were the most often not clearly described items. For example, two studies did not adjust for any confounding factors, four adjusted for them, but only one made an adjustment for fish intake among these three studies. 23 The quality assessments for the selected studies are provided in Table 1 .

**Table 1:** Methodological assessment of the selected studies

Downs and black checklist – subscales	Vigeh et al. 20	El-Badry et al. 25	Bommarito et al. 21	Liu et al. 22	Louopou et al. 23	Wang et al. 24
Reporting (10 items)	7	7	9	9	9	8
External Validity (3 items)	2	3	3	3	3	3
Internal validity – bias (7 items)	5	5	5	5	5	5
Power (1 item)	1	1	1	1	1	1
Total score	17	18	20	21	21	19

## Discussion

The present systematic review identified six studies that focused on Hg exposure and HDP, with mixed results. Previously, two systematic reviews had addressed the association of Hg exposure with blood pressure/hypertension in general population. 8 26 Together, they gathered 30 studies, but only 2 comprised pregnant women.

Very few studies have investigated the association between Hg exposure and hypertension during pregnancy and, in general, the ones that did it reported inconsistent findings. These discrepancies may partially be explained by the study methodology differences, such as sample size, exposure levels, chemical forms of Hg and its toxicokinetics, Hg biomarkers used to assess the exposure, role of metal mixture, as well as the absence or proper adjustment for confounding factors, including fish intake, a probable cause of negative confounding. 8 16 24 27 Our review also observed mixed results, with four studies reporting no association, despite the level of exposure. 20 21 22 23 The other two studies reported a positive association in groups with more exposure, although the authors used different cutoff levels for classification. 24 25 A recent systematic review with meta-analysis reported an association among those exposed to high Hg levels (hair Hg ≥ 2 µg/g) and hypertension and blood pressure. The authors suggested these levels might be considered the threshold of the toxic effect of Hg on hypertension. 8 We highlight two studies that addressed the association in both exposure scenarios, single metal, and multiple metals. 21 24 One study evaluated 28 preeclamptic women and reported no association in neither model. 21 The other investigated 854 pregnant women and found an association only in the multi-metal model (aOR multi-metal = 1.60; 95%CI = 1.08–2.38 versus aOR single metal = 1.23. 95%CI = 0.87–1.73). 24 As metals are usually dispersed in the environment, it is essential to examine their possible interactions. 28 In addition, four studies investigated the mean difference and two found greater levels in pregnant women with HDP. 23 25 However, it is pretty challenging to compare mean Hg levels between biomarkers as there is uncertainty about how mercury accumulates and is distributed across tissues. 29

Although Hg is largely distributed worldwide and hypertension is the most common medical problem encountered during pregnancy, we could retrieve only five studies for the analysis. Only one was from North Africa and none were from Latin America and the Caribbean, despite their high birth rate and low- and middle-income countries. According to 2019 data from the World Bank, 30 the fertility global tax (FGT) was 2.4 children per woman, while in the Sub-Saharan African countries, it reached 4.6. When comparing incomes, high-income countries had a FGT of 1.6, while low- and middle-income countries had 2.5 and low-income countries had 4.6. 30

All humans are exposed to some level of Hg during their lifetime. In the general population, it mainly occurs through consuming fish and shellfish contaminated with MeHg. Also, they are exposed to relatively low levels of Hg ^0^ /IHg, primarily through dental amalgam, and through inhalation from anthropogenic sources. 8 15 On the other hand, elevated exposure to Hg ^0^ /IHg is found at workplaces, such as gold mines and dentist offices. 8 In our review, most (97.4%) participants were environmentally exposed, probably through diet, although only 1 study did report its frequency, but not the type of fish. 23

The direct measurement of the level of exposure, one of the major types of biomarkers, lessens the possibility of misclassification. 31 In our review, instead of relying on the history of exposure, we chose to select studies that measured Hg levels in any biological matrix. However, we should point out the different toxicological characteristics of the three types of Hg. MeHg has a higher absorption in the gastrointestinal tract and is usually measured in blood or hair. The first indicates a recent exposure, while it points to long-term average exposure in hair. The target organ for MeHg is the brain. On the other hand, Hg ^0^ and IHg have high absorption through the respiratory system and usually are detected in urine, suggesting a recent exposure. The target organs for Hg ^0^ are the brain and kidney, and for IHg, it is the kidney. Of note, only MeHg and Hg ^0^ readily pass placental barriers, and Hg levels measured in umbilical cord blood suggest an exposure in the 3 ^rd^ trimester. 32 In our review, four studies assessed Hg exposure through blood samples (maternal blood, maternal red blood cell, and umbilical cord blood), 20 22 23 and two did it in urine samples. 21 25 Thus, we had access to information on recent exposures, not on past ones, due to the biological matrices used. 26

Overall, the selected studies were considered satisfactory according to the quality assessment tool used. As all studies were observational, confounding is potentially present. The adjustment for confounding factors was one of the items with significant gaps in our review. Two studies ignored it and four adjusted for confounding factors. Among those, only one adjusted for fish intake. 23 Fish is a food source of MeHg and essential nutrients, such as selenium and n-3 polyunsaturated fatty acids, which may have important cardiovascular benefits, such as a small but significant decline in blood pressure. 27 33 When exposure to a toxicant occurs from a food source, such as fish, negative confounding occurs, resulting in underestimating Hg toxicity and fish benefits. 27 Therefore, the four studies that did not adjust for this variable could have hampered the results.

To our knowledge, the present review was the first one to focus on the association between Hg exposure and HDP. As Hg is one of the most toxic substances widely dispersed in nature and pregnancy is a period of heightened susceptibility to environmental threats and cardiovascular risk, addressing their association is of utmost importance for public health. 16 To that end, we followed prespecified methods to review the evidence systematically. However, as a systematic review of observational studies, there are also some inherent limitations. First, the absence or no proper adjustment for confounding factors, especially fish intake, may be a significant reason the evidence is still inconclusive. Second, although we chose to accept studies that assess Hg exposure through measuring it in biological matrices (biomarkers), interindividual variations in the Hg kinetics cannot be disregarded as they are not well known. 32 Besides, using four different biomarkers (maternal blood, maternal red blood cells, umbilical cord blood, and urine) may introduce uncertainty to assess Hg exposure. Third, we observed substantial heterogeneity between the classification of groups according to Hg exposure level (low, middle, or high), even though there is a recommendation regarding human blood levels of Hg for pregnant women of up to 3.5 μg/L. 34 Fourth, we should acknowledge the lack of studies from developing countries, representing a significant gap in the literature, as populations with high fertility rates and living in low- and middle-income countries were also not investigated. Finally, we evaluated the relationship between Hg and HDP (categorical variable) but not with blood pressure levels (numerical variable). Not including the latter may lose studies addressing the Hg effect on blood pressure without necessarily leading to hypertension.

## Conclusion

Although Hg is a toxicant widely dispersed worldwide and pregnancy is a life stage of heightened susceptibility, our review retrieved only six studies addressing the association between Hg and HDP. We found mixed results, and two of these studies found a positive association in the groups with more Hg exposure. Besides, absence or no proper adjustment for confounding factors, especially the negative one (fish intake), could hamper the results. Due to the public health impact of this topic, future studies must focus on the potential effect of Hg exposure on HDP, with particular attention to adjusting for negative confounding.
